# Genetic influences on nicotinic α5 receptor (*CHRNA5*) CpG methylation and mRNA expression in brain and adipose tissue

**DOI:** 10.1186/s41021-015-0020-x

**Published:** 2015-10-01

**Authors:** Jessica E. Ramsay, C. Harker Rhodes, Keerthi Thirtamara-Rajamani, Ryan M. Smith

**Affiliations:** Center for Pharmacogenomics, The Ohio State University, Columbus, OH 43210 USA; Department of Pharmacology, The Ohio State University, 5184A Graves Hall, 333. W. 10th Ave., Columbus, OH 43210 USA; National Institute of Mental Health, Human Brain Collection Core, 10 Center Drive, Rm. 4N306, Bethesda, MD USA

**Keywords:** Nicotinic receptors, Nicotine, mRNA expression, Enhancer, Repressor, Silencer, Methylation, Epigenetics, Expression quantitative trait loci, eQTL, Functional polymorphism, Allelic expression

## Abstract

**Introduction:**

The nicotinic α5 receptor subunit, encoded by *CHRNA5*, harbors multiple functional single nucleotide polymorphisms (SNPs) that affect mRNA expression and alter the encoded protein. These polymorphisms are most notably associated with drug-taking behaviors and cognition. We previously identified common SNPs in a distant regulatory element (DRE) that increase *CHRNA5* mRNA expression in the human prefrontal cortex (PFC) and confer risk for nicotine dependence. Genome-wide epigenetic studies in PFC and adipose tissue find strong effects of the DRE SNPs on CpG methylation. However, it is unclear whether DRE SNPs influence CpG methylation *en route* to modulating *CHRNA5* mRNA expression. It is also unclear whether these polymorphisms affect expression in other brain regions, especially those mediating drug-taking behaviors.

**Results:**

By measuring total and allelic *CHRNA5* mRNA expression in human habenula and putamen autopsy tissues, we found that *CHRNA5* DRE variants considerably increase mRNA expression by up to 3.5-fold in both brain regions. Our epigenetic analysis finds no association between CpG methylation and *CHRNA5* mRNA expression in the PFC or adipose tissues.

**Conclusions:**

These finding suggests the mechanisms responsible for the genetic modulation of CpG methylation and mRNA expression are independent despite the DRE SNPs being highly associated with both measures. Our findings support a strong association between the DRE SNPs and mRNA expression or CpG methylation in the brain and periphery, but the independence of the two measures leads us to conclude that environmental factors affecting CpG methylation do not appear to directly modulate gene expression.

## Introduction

Previous studies found that allelic variation in the α5/α3/β4 neuronal nicotinic acetylcholine receptor (nAChR) subunit gene cluster on chromosome region 15q25.1 significantly increases risk for addiction to multiple classes of drugs [1–9], but confers a protective effect for cocaine addiction [8, 10]. This region also confers risk for lung cancer and chronic obstructive pulmonary disease (COPD) [2, 11–13]. A non-synonymous single nucleotide polymorphism (SNP), rs16969968, in the gene encoding the α5 subunit (*CHRNA5*) is most commonly implicated in this gene cluster. Functional analysis of this SNP suggests it reduces ligand-mediated signaling [14, 15]. In addition to rs16969968 affecting protein function, SNPs in a *cis*-acting distal regulatory element (DRE), located ~15 kb upstream of *CHRNA5*, increase mRNA expression in the prefrontal cortex (PFC) up to 4-fold [9]. This DRE harbors a cluster of six SNPs (rs7164030, rs1979905, rs1979906, rs1979907, rs880395, and rs905740) in near complete linkage disequilibrium (LD). Joint analysis of rs880395 in the DRE with rs16969968 in the Collaborative Genetic Study of Nicotine Dependence (COGEND) finds increased risk for nicotine dependence compared to risk associated with either SNP alone [9], suggesting that both SNPs can influence phenotypes associated with this genomic region.

Knockout mouse studies examining the behavioral effects of habenular and ventral tegmental area (VTA) *Chrna5* mRNA expression find that mice with a null mutation for *Chrna5* significantly increase nicotine intake [15, 16] and exhibit attenuated nicotine-induced locomotion [17]. Re-expressing *Chrna5* in the medial habenula (MHb) reduces nicotine consumption to wild-type levels [16], suggesting that α5 nAChR mRNA expression in the MHb mediates negative reward signaling through the habenulo-interpenduncular pathway. Expression of the α5 receptor subunit in GABAergic neurons of the interpeduncular nucleus (IPN) was found to further modulate this MHb output to serotonergic brain regions [18]. The medial and lateral habenula are also connected to brain regions classically associated with drug-taking behaviors that express *CHRNA5* mRNA. This includes afferent connections from the nucleus accumbens and efferent connections to the VTA and substantia nigra, which go on to innervate the PFC and striatum, respectively [19]. In the VTA, *Chrna5* modulates the sensitivity of dopaminergic neurons to acute nicotine [15] but not ethanol administration [20]. Furthermore, rs16969968 interacts with a splicing SNP in the dopamine D2 receptor gene (*DRD2*), also implicated in addiction [21], to affect multiple aspects of prefrontal cortex physiology and behavior [22]. Together, these results demonstrate a pervasive functional profile for *CHRNA5* in brain regions central to addiction and cognition. Despite strong evidence for altered *Chrna5* expression in the rodent habenula affecting addiction phenotypes, and the association of regulatory DRE SNPs with nicotine addiction, it is unknown whether the DRE SNPs affect *CHRNA5* mRNA expression in the human habenula. However, evidence that they modulate expression in the PFC, amygdala, and nucleus accumbens [5, 6, 9, 23], suggests the DRE exerts influence in cortical and subcortical brain regions.

CpG methylation in the *CHRNA5* locus is strongly influenced by the DRE polymorphisms according to genome-wide scans of *cis*-methylation quantitative trait loci (*cis*-mQTLs) in the prefrontal cortex [24] and biopsied adipose [25] tissue. Moreover, specific CpG sites within the *CHRNA5* promoter are hypermethylated in response to adverse childhood events [26]. These same adverse events confer risk for nicotine dependence, even exhibiting an *genotype x environment* interaction specifically for rs16969968 [27]. While it is reasonably hypothesized that environmental factors affect methylation, which then influences expression, this relationship has not been formally tested. Thus, the relationship between genotype, methylation, and expression remains unclear and needs to be resolved in order to identify the mechanisms underlying substance abuse with respect to *CHRNA5*.

Here, we have tested the influence of the DRE variants modulate on *CHRNA5* mRNA expression in the human habenula and putamen, by measuring total and allelic *CHRNA5* mRNA expression. We also compared expression and CpG methylation across DRE genotypes using publicly available genome-wide datasets from BrainCloud [28], BrainCloudMethyl [24], and the Multiple Tissue Human Expression Resource [25, 29]. We find the DRE SNPs modulate expression in the putamen and habenula. However, in PFC and adipose tissues where we have measures of both expression and methylation from the same individuals, we find that methylation does not appear to directly influence *CHRNA5* expression.

## Materials and methods

### Tissue samples

Twenty-one human habenula autopsy samples were dissected by a trained neuropathologist (*CRH*) or obtained from the NICHD Brain and Tissue Bank for Developmental Disorders, while 57 human posterior putamen autopsy samples were obtained through the University of Miami Brain Endowment Bank. Demographics for these human tissues are presented in Table 1. Post-mortem tissue collection was performed in accordance with local Institutional Review Board approvals. The overall study described here was performed in accordance with the Institutional Review Board of The Ohio State University.

**Table 1:** Demographic characteristics for habenula and posterior putamen tissues

Tissue (*n*)	Sex	Race^a^	Nicotine use	Cocaine use	Age (Avg. ± S.D.)	PMI (Avg. ± S.D.)	RIN (Avg. ± S.D.
Habenula (21)	Male: 12	EA: 19	Users: 5	Users: N/A	57.5 ± 18.6	35.6 ± 38.0	5.2 ± 1.4
	Female: 9	AA: 2	Non-users: 7	Non-users: N/A			
			Unknown: 9				
Putamen (55)	Male: 46	EA: 34	Users: 24	Users: 22	35.6 ± 8.9	16.7 ± 5.4	N/A
	Female: 9	AA: 10	Non-users: 31	Non-users: 33			
		Other: 11					

^a^EA: European-American, AA: African-American, Other: Mixed race and/or Hispanic

*Abbreviations: PMI* post-mortem interval, *RIN* RNA Integrity Number, as measured by Agilent Bioanalyzer 2100, *N/A* data not available

### Nucleic acid isolation & complementary DNA (cDNA) synthesis

Genomic DNA (gDNA) was isolated from all human tissues using a ‘salting out’ method adjusted for lipid-rich brain tissue, as previously described [9]. Total RNA was isolated by homogenizing the tissues in TRIzol and precipitating the RNA from the aqueous phase using isopropanol. We further purified the RNA using RNeasy Mini Kit spin columns (Qiagen, Germantown, MD) and digested latent gDNA on the column with recombinant DNaseI, as previously described [9]. cDNA preparations were made using 0.5 μg total RNA for each sample. Gene-specific primers (25 nM) supplemented with Oligo-dT (5 μM) were used to prime the reverse transcription reaction.

### Sample genotyping

SNPs rs16969968, rs615470, and rs7164030 were genotyped by restriction fragment length polymorphism (RFLP) methods. rs16969968 and rs615470 serve as marker SNPs for measuring allelic mRNA expression, which were chosen because of their high minor allele frequencies, high likelihood to be present in the mature mRNA, and LD pattern which suggests their minor alleles are present on different haplotypes. rs7164030 serves as the representative marker of DRE, which include 5 additional SNPs in high LD (rs1979907, rs1979906, rs1979905, rs880395, and rs905740). The gDNA regions surrounding these SNPs were amplified using primers tagged with a fluorophore (6-FAM or HEX) and the resultant amplicons were cut with restriction enzymes (rs16969968-Taq1a; rs615470-CviQI; rs7164030-Tsp509I) that recognize only one of the two alleles resulting from the presence of the polymorphism. Fragments were resolved on an ABI 3730 DNA Analyzer (Life Technologies) or by standard gel electrophoresis (1.5 % agarose).

### Total and allelic mRNA expression measurement

Total *CHRNA5* and β-actin (*ACTB*) mRNA expression was measured in all human and mouse tissues by qPCR using an ABI 7500 Fast Sequence Detection System (Life Technologies). In addition, we measured the expression of two highly-enriched habenula markers, *POU4F1* and *CHRNB3*, in the habenula samples to determine the purity of the dissections. The relative quantity of *CHRNA5* mRNA was normalized within each sample to *ACTB* mRNA expression for statistical analysis. The influences of available covariates (age, sex, race, post-mortem interval, RNA integrity number, nicotine use, or habenula purity) were tested on *ACTB*-normalized total *CHRNA5* mRNA expression in each brain region using stepwise linear regression.

We quantified allelic mRNA expression in habenula and putamen samples heterozygous for rs16969968 or rs615470 using a fluorescent primer extension method (SNaPshot), as previously described [9]. Fluorescently-labeled primer extension fragments, representing the two different alleles of rs16969968 or rs615470, were resolved on an ABI 3730. The fluorescent peak heights for each allele, determined using GeneMapper 4.0 Software (Life Technologies), were used to calculate relative allelic expression ratios (ancestral/variant allele). For each sample, at least two separate measurements were used to calculate allelic expression imbalance (AEI). Allelic ratios for cDNA were normalized against the overall average ratio calculated for gDNA for each marker SNP. We subsequently compared the absolute magnitude of the allelic expression in samples heterozygous for rs7164030 *versus* homozygotes for either allele.

### CHRNA5 mRNA expression and CpG methylation using BrainCloud and MuTHER

We utilized existing repositories of transcriptome-wide mRNA expression, genome-wide methylation profiles, and SNP genotypes in postnatal PFC (BrainCloud; http://braincloud.jhmi.edu/) and adipose tissue (MuTHER; http://www.muther.ac.uk/) to test interactions between DRE SNPs, expression, and CpG methylation. Detailed methods for these studies are available in their primary publications [24, 25, 28, 29]. Source data can be obtained from the Gene Expression Omnibus (Accession: GSE30272), dbGAP (Accession: phs000417), EMBL-EBI ArrayExpress (Accessions: E-TABM-1140 and E-MTAB-1866), and by applying to the TwinsUK Consortium (http://www.twinsuk.ac.uk/).

Briefly, samples were genotyped with a variety of Illumina arrays (HumanHap300, HumanHap610Q, HumanHap650Y, Human 1 M-Duo, and Human 1.2 MDuo 1 M) and imputed to 1000 Genomes populations using IMPUTE2. From the imputed data, we used rs7164030 as a surrogate marker of the DRE to test genetic effects on expression and methylation. Samples were also measured for genome-wide CpG methylation, using the Infinium HumanMethylation450k BeadChip assay, and transcriptome-wide mRNA expression, using Illumina 49 K Oligo Arrays (BrainCloud) or HumanHT-12 v3 BeadChips (MuTHER). For analyses, we used CpG probes cg22563815 and cg17108064, which measures methylation at CpG sites 913 and 802 nucleotides upstream of the annotated *CHRNA5* gene (hg19 chr15: 78856949 and chr15:78857060), respectively. Although these CpG probes are ~12 kb downstream from the DRE SNPs, they are among the highest scoring mQTLs for the DRE SNPs in the *CHRNA5* gene region. For expression, we used probes hHC002196 (BrainCloud) and ILMN_1770044 (MuTHER), which hybridize to *CHRNA5* mRNA in the 3′ untranslated region and exon 5, respectively.

### Statistical analyses

Statistical analyses were performed in *R* (x64 v.3.1.0) with standardized β-coefficients calculated by the QuantPsyc package. For all datasets, we used interquartile range (IQR) to exclude extreme outliers, defined as data points below $Q1 - 3 xIQR$ or above $Q3+3 xIQR$. Next, we identified significant covariates using stepwise linear regression and AIC, using the [step] function to reach a minimal adequate model. We subsequently included significant covariates in analyses of rs7164030 genotype on expression and methylation or as interaction terms in linear regression models of expression and methylation. Potential covariates in our putamen and habenula expression datasets included age, sex, race, smoking history, cocaine use, and post-mortem interval. Potential covariates in the BrainCloud data included sex, age, race, and an estimate of neuron enrichment that was specific to methylation data [30]. Age and batch-specific effects were considered as a potential covariate in the MuTHER dataset. We tested for an overall effect of methylation on expression using linear regression across the entire BrainCloud or MuTHER sample populations and report the standardized β-coefficient for the methylation measure. We further tested this relationship within each rs7164030 genotype group to identify any genotype-specific effect that could be obscured when examining the population as a whole.

## Results

### Total and allelic CHRNA5 mRNA expression in putamen and habenula

**Fig. 1:**
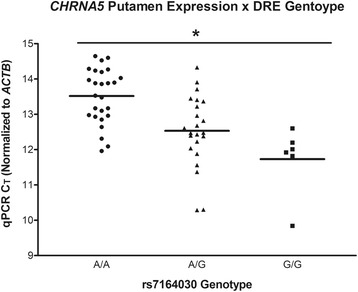
*CHRNA5* mRNA expression in the putamen compared across rs7164030 genotypes, as measured by qPCR. Samples homozygous for the major “A” allele (A/A) of rs7164030 have approximately 3.5-fold less *CHRNA5* mRNA versus samples homozygous for the minor “G” allele, with heterozygotes exhibiting intermediate levels (ANOVA *p*=1.82×10^−6^)

Two putamen samples were excluded from analyses due to poor RNA quality as indicated by *ACTB* expression >2 standard deviations above the mean of the remaining samples, leaving 55 total putamen samples. Stepwise linear regression revealed sex as a significant covariate of *CHRNA5* mRNA expression measured via qPCR in the putamen. We found a significant effect of the representative DRE SNP rs7164030 genotype on putamen expression (*n*=55, *F*=28.90, *p*=1.82×10^−6^; Fig. 1), whereby homozygous minor “G” allele samples expressed 3.5-fold more *CHRNA5* mRNA than homozygous major “A” allele samples, consistent with our previous findings in PFC [9]. Examining the influence of rs7164030 on habenular *CHRNA5* mRNA expression via qPCR with race as a significant covariate revealed no significant effect of genotype, although the direction of the genotypic effect is consistent with our findings in the putamen and PFC. We also noted that the purity of the habenula dissection, as determined by *POU4F1* or *CHRNB3* expression, did not influence *CHRNA5* mRNA expression. A comparative analysis of habenula *CHRNA5* mRNA expression with previously measured PFC expression found no enrichment in the habenula relative to the PFC in humans, consistent with previous reports of generally low expression in these areas in rodents [18, 31].

**Fig. 2:**
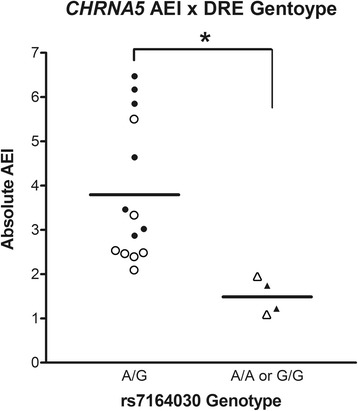
Absolute allelic expression imbalance in putamen (filled markers) and habenula (open markers) compared across rs7164030 genotype. Samples heterozygous for rs7164030 exhibit significantly greater AEI than samples homozygous for rs7164030 (ANOVA *p*=0.012), consistent with the expectation that AEI is observed in samples heterozygous for the functional allele. Samples homozygous for either DRE allele are not expected to exhibit AEI

We measured allelic mRNA expression in 21 of 55 putamen samples heterozygous for either rs16969968 or rs615470 (9 co-heterozygous) and 9 of 21 habenula samples heterozygous for rs16969968. The low expression of *CHRNA5* in both tissues required us to average the allelic ratio measurements at the two marker SNPs, as done for the putamen, or take an increased number of measurements at the same SNP, as done for the habenula. Thus, the averaged data is only presented for the 9 co-heterozygous putamen samples, while the data for all habenula samples is presented for marker SNP rs16969968. Samples heterozygous for rs7164030 exhibited AEI ranging from 2.1 to 6.5-fold differences between the expression of the two alleles, while samples homozygous for either allele of rs7164030 all displayed AEI of <2-fold, consistent across both brain regions. We observed greater expression for the major allele of rs16969968 relative to the minor allele in all but one sample exhibiting >2-fold AEI, consistent with the major allele for rs16969968 residing on the high expressing DRE haplotype. Comparing the absolute magnitude of AEI across rs7164030 genotype, we find heterozygotes exhibit significantly greater AEI versus homozygotes (*F*=7.99, *p*=0.012; Fig. 2), supporting the hypothesis that the DRE SNPs exert their function in both the habenula and putamen.

### DRE SNPs, CHRNA5 expression, and methylation in BrainCloud and MuTHER

**Fig. 3:**
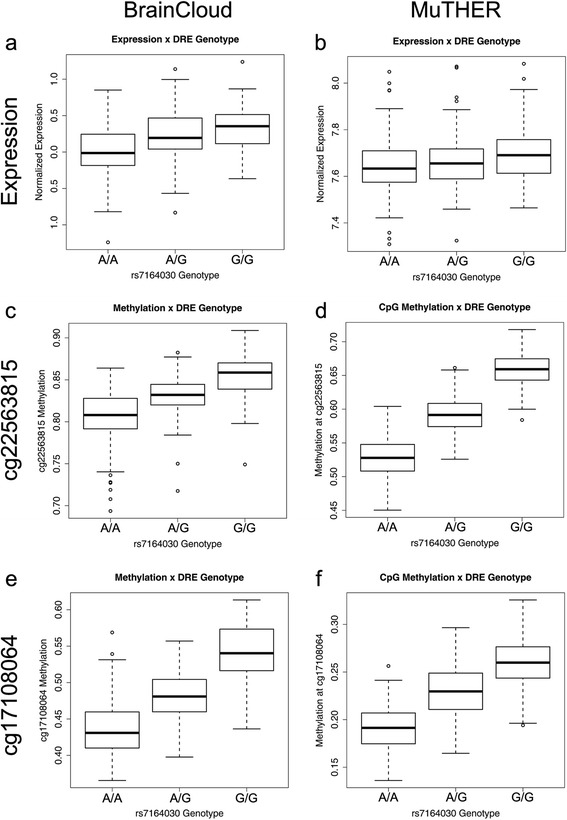
Expression and methylation across rs7164030 genotype in BrainCloud and MuTHER. The major “A” allele of rs7164030 was significantly and consistently associated with lower *CHRNA5* mRNA expression in the BrainCloud (**a**) and MuTHER (**b**) datasets. The “A” allele was also associated with lower CpG methylation measured at two different probes (cg22563815 and cg17108064) in the BrainCloud prefrontal cortex (**c** and **e**) and the MuTHER adipose tissue (**d** and **f**)

*CHRNA5* mRNA expression differed significantly across rs7164030 genotype in the BrainCloud dataset (*F*=28.57, *p*=2.30×10^−7^; age and sex included as significant covariates). Consistent with our previous study in the PFC [9], samples homozygous for the variant G allele of rs7164030 expressed 3.67-fold more *CHRNA5* mRNA than samples homozygous for the ancestral A allele (Fig. 3a). rs7164030 genotype was also significantly associated with *CHRNA5* expression in MuTHER (*F*=13.06, *p*=3.28×10^−4^; Fig. 3b).

Methylation in the BrainCloud dataset significantly differed across rs7164030 genotype in the PFC for both CpG probes (cg22563815: *F*=109.17, *p*=5.87×10^−21^; race, age, and neuron enrichment estimate as significant covariates; cg17108064: *F*=191.06, *p*=1.77×10^−31^; race as a significant covariate). Here, samples homozygous for the variant G allele of rs7164030 had significantly greater CpG methylation at both probes relative to the ancestral A allele homozygotes (Fig. 3c and e). Methylation was also significantly higher across both probes for the variant G allele carriers in the MuTHER dataset (cg22563815: *F *= 1703.48, *p*=5.54×10^−172^; bisulfite conversion gDNA concentration and efficiency included as covariates; cg17108064: *F*=614.33, *p*=3.73×10^−92^; batch and bisulfite conversion efficiency included as covariates; Fig. 3d and f).

**Fig. 4:**
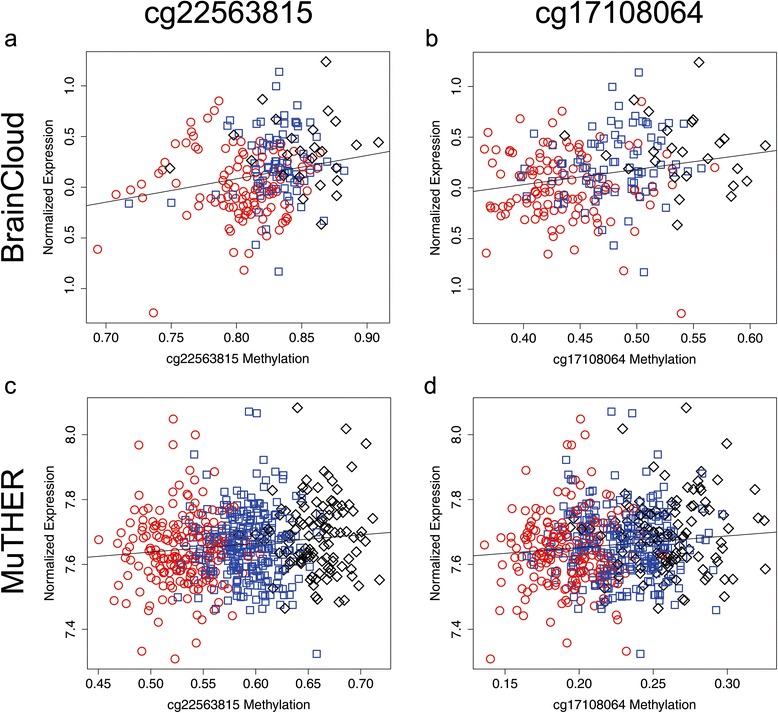
Scatterplots for CpG methylation and *CHRNA5* mRNA expression in BrainCloud and MuTHER. *CHRNA5* expression is moderately correlated with CpG methylation measured in the BrainCloud prefrontal cortex data by probes cg22563815 (**a**) and cg17108064 (**b**). Similar results were obtained for the same probes in the MuTHER adipose tissue (**c** and **d**). However, the correlation between expression and CpG methylation is explained by rs7164030 genotype, apparent by the stratification of the genotype groups in the scatterplots (A/A = red circles, A/G = blue squares, G/G = black diamonds). Furthermore, linear regression performed within each genotype group finds no significant relationship between methylation and expression, arguing against direct modulation of expression by methylation

Because the DRE SNPs are associated with significant increases in both expression and methylation, we further asked if the increased expression can be predicted by greater methylation using linear regression. In both the BrainCloud and MuTHER datasets, we found that methylation at either probe did not significantly predict *CHRNA5* expression. The linear model for cg22563815 in BrainCloud (Expression ~ sex + cg22563815*methylation*race*age*neuron count estimate*rs7164030) found no significant effect for methylation at cg22563815 (*t* = 0.42, *p*=0.67 β=0.30; Fig. 4a). Similarly, the model for cg17108064 (Expression ~ sex + age + cg17108064*race*rs7164030) also revealed no significant effect for methylation (*t*=−0.84, *p*=0.40 β=−0.11; Fig. 4b). In the MuTHER dataset, the linear model for methylation at cg22563815 (Expression ~ cg22563815*bisulfite conversion concentration*bisulfite conversion efficiency*rs7164030) showed a trend towards significance (*t*=−1.95, *p*=0.051, β=−2.34; Fig. 4c), while methylation at cg17108064 (Expression ~ cg17108064*batch*bisulfite conversion efficiency*rs7164030) did not predict expression (*t*=1.48, *p*=0.14, β=0.99; Fig. 4d).

Evident in each of our linear models is the confounding influence of genotype on both methylation and expression (Fig. 4). Although we accounted for this influence statistically in the model examining all samples, we subsequently tested whether it was still possible for methylation to influence expression on specific genetic backgrounds (*i.e.* if one were to carry the DRE SNPs), which could be obscured in the full linear model. Thus, we performed linear regression within each of the rs7164030 genotype groups, finding no evidence that methylation at either probe affects expression in any of the genetic backgrounds defined by the DRE SNPs (Table 2).

**Table 2:** Linear regression results for CpG methylation and *CHRNA5* gene expression across rs7164030 genotypes

Dataset	CpG probe	rs7164030 genotype (n)	Standardized β coefficient for methylation	*t*-value	*p*-value
BrainCloud	cg22563815	A/A (118)	0.267	0.363	0.717
	cg22563815	A/G (77)	1.166	0.390	0.698
	cg22563815	G/G (25)	−16.837	−0.397	0.700
	cg17108064	A/A (118)	−0.171	−1.424	0.157
	cg17108064	A/G (77)	0.153	1.072	0.288
	cg17108064	G/G (25)	0.024	−0.040	0.969
MuTHER	cg22563815	A/A (184)	4.069	0.528	0.598
	cg22563815	A/G (270)	0.010	0.014	0.989
	cg22563815	G/G (111)	−2.964	−1.718	0.089
	cg17108064	A/A (184)	0.981	0.398	0.691
	cg17108064	A/G (270)	−1.288	−0.813	0.417
	cg17108064	G/G (111)	−2.510	−0.639	0.524

## Discussion

Our findings reveal pervasive influence of the DRE SNPs on *CHRNA5* mRNA expression and methylation in brain and adipose tissue, whereby the minor DRE alleles are associated with greater expression and methylation. This genotypic difference is consistent with our findings in the PFC for both the BrainCloud dataset and in our previous study [9]. The habenula did not show a main effect of the DRE SNPs on total *CHRNA5* expression, but we observed strong allelic differences in the habenula that perfectly correlate with the DRE SNPs, consistent with the interpretation that they modulate *CHRNA5* expression in the habenula. While significant, the influence of the DRE SNPs on *CHRNA5* expression is not as strong in adipose tissue. We previously reported no influence of the DRE SNPs in lymphoblastoid cell lines (LCLs), but other studies with larger sample sizes have found *cis*-eQTLs for *CHRNA5*, implicating the DRE SNPs in peripheral whole blood [32], monocytes [33], and lung [34]. Thus, it is likely that the DRE SNPs are modulating expression of *CHRNA5* in peripheral tissues, but exert less influence relative to their impact in the brain.

The location and epigenetic histone markings in the *CHRNA5* locus harboring the DRE SNPs previously led us to propose they act in an enhancer [9]. Data from the ENCyclopedia Of DNa Elements (ENCODE) Project [35] viewed on the UCSC Genome Browser [36] shows histone modifications in a lymphocyte cell line (GM12878) consistent with enhancers, including histone 3 lysine 4 monomethylation (H3K4Me1) and light trimethylation (H3K4Me3), and H3 lysine 27 acetylation (H3K27Ac). However, when a portion of the DRE containing rs880395, rs905740, and rs7164030 was sub-cloned into a vector upstream of a minimal promoter, it acted as a repressor, with no significant expression differences between DRE haplotypes [23]. Given these contradictory results, we find it possible that the DRE contains both enhancer and repressor elements. A dual enhancer/repressor mechanism is not novel. Perhaps the most well-known example is the RE-1 Silencing Transcription Factor (REST), which silences neuronal genes in the periphery [37], but has the ability to enhance gene expression in the brain [38, 39]. Evolutionary studies of *cis*-acting enhancer elements supports the possibility that multiple variants affecting enhancer function can arise together within a population to high frequency [40], sometimes co-opting cryptic or existing regulatory sequences to derive their new functions [41], as we would assume occurred for the DRE SNPs in *CHRNA5*. A more thorough analysis of the evolutionary constraints on the *CHRNA5* locus could provide clues about the adaptive evolution of regulatory elements in humans.

In addition to the epigenetic histone modifications present in the *CHRNA5* locus, CpG methylation is strongly associated with *CHRNA5* SNPs. Since CpG methylation can repress transcription [42] it led us to examine *CHRNA5* CpG methylation and mRNA expression using BrainCloud and MuTHER. Because the DRE SNPs were strongly associated with increased methylation and expression in both datasets, we expected methylation to be positively correlated with expression, thus providing mechanistic evidence linking epigenetic modulation of the *CHRNA5* locus and gene expression. Instead, we found that methylation and expression were independent when accounting for DRE genotype and other significant covariates. Evidence that *CHRNA5* expression and methylation are independent is important for delineating mechanisms underlying drug addiction that is associated with this gene locus, since both methylation and expression apparently influence addiction risk. One explanation that unifies expression and methylation, that also serves as a caveat of this study, is that methylation influences the expression of *CHRNA5* in a way that was not detected here. Such scenarios could include changes in alternative splicing or transcription start site usage which do not change the overall levels of *CHRNA5* mRNA, but alter the makeup of the mRNA. The GENCODE project has annotated an alternatively spliced transcript (ENST00000559554.1), but it has only been observed as an expressed sequence tag in a neuroblastoma cell line.

Methylation at the *CHRNA5* locus is sensitive to environmental factors, as demonstrated by childhood adverse events (CA). CA results in hypermethylation and increased risk for drug dependence [26, 27]. Males carrying rs16969968 who experience CA are at greater risk for dependence relative to those without rs16969968 [27]. In the context of our findings, we do not find it likely that hypermethylation changes mRNA expression, although we cannot rule out that CA-induced hypermethylation can be much greater than observed in our samples and subsequently affect expression. Reanalyzing existing CA studies to include the DRE SNPs in addition to rs16969968 could shed some light on the relationships between CA, methylation, and smoking risk. However, a study examining environmental factors, CpG methylation, mRNA expression, and drug dependence would be ideal for resolving the risk conferred by 15q25.1.

Finally, the strong impact of *Chrna5* expression in mouse MHb and VTA on nicotine consumption despite low levels of mRNA expression in mice and humans signifies the importance of the specific cell types on which these receptors are expressed. The MHb afferents that express the α5 subunit project to the interpeduncular nucleus [19], which also contains GABAergic neurons expressing the α5 subunit [18], modulating aversiveness associated with nicotine intake [16] and withdrawal [43]. Dopaminergic neurons in the VTA express the α5 receptor subunit [44] and project to multiple addiction-related brain regions, including the cortex and insula via the mesocortical pathway and limbic areas, the nucleus accumbens, lateral habenula, and amygdala through the mesolimbic pathway [45]. Finding ways to modulate the firing of the cells expressing *CHRNA5*, directly or indirectly, without targeting nicotinic α5-containing receptors could provide avenues for treating addictive behaviors that circumvent the inherent challenges of developing small molecules for nicotinic receptors. Identifying promising new targets will require a firm understanding of addiction neurocircuitry and of genetic expression within specific cell types in the habenula, IPN, and VTA, in order to exploit the aversive signaling properties of these cells in the context of drug abuse.

## Conclusions

Our findings support pervasive but independent influence of the *CHRNA5* DRE SNPs on mRNA expression and CpG methylation in the brain and periphery. With evidence that environmental influences modulate CpG methylation in this region, we advocate for future studies to incorporate environmental, epigenetic, and genetic factors in the pathogenesis of addiction associated with *CHRNA5*. Finally, clinical and molecular genetic studies of the *CHRNA5/A3/B4* locus suggest the presence of unaccounted for functional regulatory variants [46], requiring additional studies to delineate the exact functional variant(s) and their phenotypic impact to further disentangle disease risk conferred by 15q25.1.
